# Overexpression of SERBP1 (Plasminogen activator inhibitor 1 RNA binding protein) in human breast cancer is correlated with favourable prognosis

**DOI:** 10.1186/1471-2407-12-597

**Published:** 2012-12-13

**Authors:** Nuran Bektas Serce, Andreas Boesl, Irina Klaman, Sonja von Serényi, Erik Noetzel, Michael F Press, Arno Dimmler, Arndt Hartmann, Jalid Sehouli, Ruth Knuechel, Matthias W Beckmann, Peter A Fasching, Edgar Dahl

**Affiliations:** 1 Department of Pathology, University Hospital Bonn, Sigmund-Freud-Str. 25, 53127, Bonn, Germany; 2 Department of Pathology, Vorarlberger Krankenhaus-Betriebsgesellschaft m.b.H. Hospital, Feldkirch, Austria; 3 Signature Diagnostics AG, Voltaireweg 4b, 14469, Potsdam, Germany; 4 Molecular Oncology Group, Institute of Pathology, University Hospital of the RWTH Aachen, Pauwelsstrasse 30, 52074, Aachen, Germany; 5 Department of PathologyKeck School of Medicine, University of Southern California/Norris Comprehensive Cancer Center, 1441 Eastlake Avenue, Los Angeles, CA, 90033, U.S.A; 6 Department of Pathology, St.-Vincentius-Kliniken, Südenstr. 37, 76137, Karlsruhe, Germany; 7 Department of Pathology, University Hospital Erlangen, Krankenhausstrasse 12, 91054, Erlangen, Germany; 8 Department of Gynaecology, Charité Campus Virchow-Klinikum, Augustenburger Platz 1, 13353, Berlin, Germany; 9 Department of Gynaecology, University Hospital Erlangen, Universitätsstrasse 21-23, 91054, Erlangen, Germany; 10 David Geffen School of Medicine, Division of Hematology and Oncology, University of California at Los Angeles, Los Angeles, USA

**Keywords:** SERBP1, uPA/PAI-1, Gene expression, Breast cancer, Prognostic marker

## Abstract

**Background:**

Plasminogen activator inhibitor 1 (PAI-1) overexpression is an important prognostic and predictive biomarker in human breast cancer. SERBP1, a protein that is supposed to regulate the stability of *PAI*-*1* mRNA, may play a role in gynaecological cancers as well, since upregulation of SERBP1 was described in ovarian cancer recently. This is the first study to present a systematic characterisation of SERBP1 expression in human breast cancer and normal breast tissue at both the mRNA and the protein level.

**Methods:**

Using semiquantitative realtime PCR we analysed SERBP1 expression in different normal human tissues (n = 25), and in matched pairs of normal (n = 7) and cancerous breast tissues (n = 7). SERBP1 protein expression was analysed in two independent cohorts on tissue microarrays (TMAs), an initial evaluation set, consisting of 193 breast carcinomas and 48 normal breast tissues, and a second large validation set, consisting of 605 breast carcinomas. In addition, a collection of benign (n = 2) and malignant (n = 6) mammary cell lines as well as breast carcinoma lysates (n = 16) were investigated for SERBP1 expression by Western blot analysis. Furthermore, applying non-radioisotopic in situ hybridisation a subset of normal (n = 10) and cancerous (n = 10) breast tissue specimens from the initial TMA were analysed for SERBP1 mRNA expression.

**Results:**

SERBP1 is not differentially expressed in breast carcinoma compared to normal breast tissue, both at the RNA and protein level. However, recurrence-free survival analysis showed a significant correlation (*P* = 0.008) between abundant SERBP1 expression in breast carcinoma and favourable prognosis. Interestingly, overall survival analysis also displayed a tendency (*P* = 0.09) towards favourable prognosis when SERBP1 was overexpressed in breast cancer.

**Conclusions:**

The RNA-binding protein SERBP1 is abundantly expressed in human breast cancer and may represent a novel breast tumour marker with prognostic significance. Its potential involvement in the plasminogen activator protease cascade warrants further investigation.

## Background

SERBP1 (PAI-RBP1) is a *PAI1* (plasminogen activator inhibitor 1) *mRNA* binding protein encoded by the *SERBP1* gene localised on chromosome 1p31 [1]. SERBP1 is supposed to regulate the stability of *PAI1 mRNA* by binding to its cyclic nucleotide-responsive sequence (CRS) [2]. Interestingly, it has been found that binding of SERBP1 protein to *PAI1 mRNA* may either stabilise or destabilise the PAI-1 transcript depending on the intracellular localisation of SERBP1 [2]. Cyclic nucleotides are thought to control this process by recruiting SERBP1 to the nucleus if the protein acts to stabilise or to the cytoplasm if it acts to destabilise *PAI1 mRNA*[2]. Yet the exact molecular mechanisms of this dual behaviour have not been elucidated [3]. SERBP1 was also assigned a role in chromatin architecture as it was found to interact with the C-terminal region of the human chromatin remodelling factor CHD-3 (chromo-helicase-DNA-binding domain protein-3) [4]. CHD proteins are members of the chromodomain family, a class of proteins which are involved in chromatin remodelling and transcriptional regulation [4]. Clearly, chromatin remodelling is a key process in the regulation of gene expression since it affects the DNA’s tertiary and higher order structures including nucleosome packing, the formation of DNA loops or its supercoiling [5]. Furthermore, SERBP1 is known to mediate non-nuclear progesterone effects by binding to PGRMC1 (progesterone receptor membrane component-1) which is involved in mediating antiapoptotic actions of progesterone [6].

As above mentioned, SERBP1 is a binding protein of *PAI1 mRNA*[1]. Both PAI-1 (plasminogen activator inhibitor 1) and uPA (urokinase-type plasminogen activator) are part of the plasminogen activator (PA) system playing a central role in physiological processes including fibrinolysis, angiogenesis and wound healing as well as in tumour cell invasion and metastasis [7,8]. Plasminogen activation results in the formation of plasmin, a serine protease, which activates the proenzyme uPA to the proteolytic active uPA and is also the activator of several matrix metalloproteinases leading to degradation of the extracellular matrix and basement membrane [7,8]. Overexpression of uPA and PAI-1 has been found in malignant solid tumours in a variety of human cancers, including ovarian and breast cancer [7-9]. High protein levels of uPA and PAI-1 are associated with poor prognosis in lymph node-negative breast cancer patients and are established predictive factors in clinical practice [10,11]. In ovarian cancer, overexpression of uPA and PAI-1 was correlated with poor clinical outcome as well [12,13]. Recently, overexpression of the *PAI1 mRNA* binding protein (SERBP1) was also detected in ovarian cancer where it significantly correlated with advanced tumour stage [1]. In human breast cancer expression of SERBP1 has not been examined so far. To our best knowledge, this is the first study that systematically analysed the expression of SERBP1 in human breast carcinomas and normal breast tissues both at the mRNA and the protein level. We analysed the results especially in correlation to clinicopathological data like hormone receptor status, HER2 status and patient survival in order to validate SERBP1 as a new prognostic marker and potential drug target in the treatment of human breast cancer.

## Methods

SERBP1 protein expression in breast cancer patients was assessed using two independent breast cancer cohorts on TMAs. The first tumour cohort has been previously described [14,15] and consisted of 193 breast cancer specimens and 48 normal breast tissue specimens. The TMA contained one tissue core from non-selected, formalin-fixed and paraffin-embedded primary breast cancer specimens diagnosed between 1994 and 2002 at the Institute of Pathology, University of Regensburg, Germany. Patients’ age ranged from 25 to 82 years with a median age of 56 years. An experienced surgical pathologist (A.H.) evaluated H&E-stained slides of all specimens prior to construction of the TMA in order to identify representative tumour areas. Histologically, all tumours were graded according to Elston and Ellis [16]. Clinical follow-up data, provided by the Central Tumour Registry, Regensburg, Germany were available for all 193 breast cancer patients with a median follow-up period of 78 months (range 0-148 months).

The second TMA for validation consisted of 967 breast specimens of which 605 breast carcinomas were analysable. The second TMA was constructed from a cohort of breast cancer patients who were participants of a case control trial for the assessment of breast cancer susceptibility markers and prognostic factors, the Bavarian Breast Cancer Cases and Controls Study (BBCC), which has been described previously, including methods for the data collection [17,18]. Briefly, database closure for this analysis was December 31, 2008 with a median follow up of 5.3 years. Of 967 BBCC patients a paraffin embedded tissue was available. A 0.6 mm punch was retrieved from the breast cancer tumour after a pathologist (A.D.) evaluated H&E-stained slides of all specimens. Data concerning the hormone receptor status were obtained from original pathological reports of these specimens; the other data were collected from the patients’ medical records and an epidemiological questionnaire which was completed in a personal interview.

All patients included in this study gave informed consent for further analysis of their tissue for research purposes and publication of the data. The Instructional Review Board of the participating centre approved the study (Ethics Committee of the Medical Faculty of the Friedrich-Alexander University of Erlangen, reference number: 2700; Ethics Committee of the Medical Faculty of the University of Aachen (reference number: 206/09) and Regensburg).

### Benign human tissue mRNAs

For RT-PCR analysis commercially available mRNAs (Clontech, Germany) derived from different benign human organs (n = 25) were used. Tissue included in this selection were skeletal muscle, heart, spinal cord, brain, thymus, kidney, colon, bone marrow, placenta, adrenal gland, prostate, stomach, trachea, small intestine, uterus, thyroid, lymph node, pituitary gland, mammary gland, spleen, pancreas, cervix, salivary gland, testis and liver.

### Cryoconserved human breast cancer samples

For Western blot analysis we used lysates of cryoconserved human breast cancer tissues with low (n = 8) and high (n = 8) levels of PAI-1 protein. The PAI-concentrations were measured by application of a commercially available ELISA-test (FEMTELLE®). As established in clinical routine, a PAI-1 level <14 ng/mg total protein was defined as low and >14 ng/mg total protein as high [10,11]. Additionally we analysed cryoconserved matched pairs of normal (n = 7) and cancerous (n = 7) breast tissue samples for mRNA expression analysis.

### Cell lines

The human mammary epithelial cell lines MCF12A and MCF10A as well as the breast cancer cell lines ZR75-1, BT20, HS578T, SKBR3, MDA-MB468 and MDA-MB231 were obtained from the ATCC (Rockville, MD, USA) and cultured as previously described [19].

### RNA extraction and reverse transcription

Total RNA was isolated by use of TRIzol reagent (Invitrogen, Carlsbad, CA, USA) according to the manufacturers’ recommendations. Of the obtained RNA, 1 μg was reverse transcribed using the Reverse Transcription System (Promega, Madison, WI, USA). In order to improve transcription rate we mixed oligo-dT and pdN_6_ primers 1:1.

### Semiquantitative realtime PCR

Semiquantitative PCR was performed using the LightCycler system together with the LightCycler DNA Master SYBR Green I Kit (Roche Diagnostics, Mannheim, Germany) [19]. To ensure experiment accuracy, all reactions were performed in triplicate. Primer sequences were: *SERBP1* sense 5^′^- CCA CCT CGT GAA CGA AGA TT -3^′^ and antisense 5^′^- ACC ACC ACG ACC TCG AAT AG -3^′^; *GAPDH* sense 5^′^- GAA GGT GAA GGT CGG AGT CA -3^′^ and antisense 5^′^- AAT GAA GGG CTC ATT GAT GG -3^′^. Annealing temperatures for both genes were set to 60°C. Reaction specificity was controlled by post-amplification melting curve analyses as well as by gel electrophoresis of the obtained products.

### Non-radioisotopic in situ hybridisation

Non-radioisotopic RNA in situ hybridisation [20,21] was used to analyse the mRNA expression of SERBP1. In short, cRNA sense and antisense probes were transcribed from linearised SERBP1 cDNA clone AA164643 using an in vitro transcription kit (Roche Diagnostics, Mannheim, Germany) according to the instructions of the manufacturer. The size of the in vitro transcribed RNA was confirmed by agarose gel electrophoresis. Paraffin embedded tissue sections were deparaffinised, rehydrated and fixed prior to hybridisation. Hybridisation of sections was performed in hybridisation buffer containing 2.5 ng/μl Digoxigenin-labeled RNA probes for 12 h at 65°C. After stringent washes to remove excess probe, the slides were incubated with the anti-DIG-AP antibody (Roche Diagnostics, Mannheim, Germany) for 12 h at 4°C in order to visualise the hybridised transcripts. The slides were then rinsed for 2–4 days in washing buffer. For detection of hybridisation signals, slides were incubated in 1 ml BM-Purple (Roche Diagnostics, Mannheim, Germany) containing 2 mM Levamisol and 0.1% Tween-20. Sections were counterstained with Texas Fast Red (Sigma, Munich, Germany).

### Immunohistochemistry

Immunohistochemical (IHC) studies for the expression of HER2 utilised an avidin-biotin peroxidase method with a 3,3^′^-diaminobenzidine (DAB) chromatogen. After antigen retrieval (microwave oven for 30 min at 200 W) IHC was carried out in a NEXES immunostainer (Ventana, Tucson, AZ, USA) following the manufacturer’s instructions. The following primary antibodies were used: anti-HER2 (DAKO, Hamburg, Germany; 1:400), anti-ER and anti-PR (Novocastra, Newcastle Upon Tyne, UK; 1:20). For target proteins the ChemMate detection kit (DAKO) was used. A surgical pathologist (A.H.) performed a blinded evaluation of the TMA slides without knowledge of clinical data. Causes of non-interpretable results included lack of tumour tissue and presence of necrosis or crush artefacts. HER2 expression was scored according to the DAKO HercepTest. For the evaluation of ER and PR presence, a semiquantitative immunoreactivity score (IRS), as described by Remmele and Stegner [22], was used considering staining intensity and percentage of positive cell nuclei. The staining intensity was described by scores between 0 and 3 (0 = no reaction, 1 = low, 2 = moderate, 3 = strong). Accordingly, the number of positive cell nuclei was counted and scored between 0 and 4 (0 = no positive cell nuclei, 1 = < 10% positive cell nuclei, 2 = 10-50% positive cell nuclei, 3 = 51-80% positive cell nuclei, 4 = > 80% positive cell nuclei). The product of staining intensity and percentage of positive cell nuclei resulted in a score (IRS) between 0 and 12. Each sample was categorised by this rating score. Immunohistochemical evaluation of nuclear and cytoplasmic staining for SERBP1 was done equivalently [22].

### Generation of a polyclonal SERBP1 antibody

As we started our investigations commercial polyclonal SERBP1 antibodies which were applicable for immunohistochemistry on paraffin embedded tissues were not available. Therefore we commissioned Eurogentec Inc. (Liège, Belgium) to produce a polyclonal SERBP1 antibody. Eurogentec gained the SERBP1 antibody by immunisation of rabbits with the corresponding peptide (peptide sequence: CKKEGIRRVGRR). Specificity was proven by a peptide competition experiment using Western blot analysis (Additional file 1: Figure S1).

### SERBP1 Immunohistochemistry

The tissue microarrays – both, the evaluation set and the validation set - were subjected to immunostaining using the Advance Kit (DAKO K4068) following the manufacturer’s instructions. Paraffin-embedded breast carcinomas served as positive controls. After deparaffinisation and rehydration the tissue samples were boiled in a microwave oven for 30 min at 200 W in 10 mM sodium citrate buffer (pH 7.2). Endogenous peroxidase was blocked by peroxidase blocking solution (DAKO S2023) for 5 min. The polyclonal primary antibody SERBP1 (Eurogentec, EP060994, rabbit, Liège, Belgium) was applied (1:40 dilution) for 30 min at room temperature. In negative controls the primary antibody was omitted. For signal detection 3,3^′^-diaminobenzidine (DAB) was used. Slides were counterstained with hematoxylin and after dehydration mounted in Vitro-Clud (Langenbrinck, Emmendingen, Germany). Two experienced pathologists (N.B.S., A.H.) scored the immunohistochemical staining intensity according to the scoring system suggested by Remmele and Stegner [22].

### SERBP1 Western blot analysis

Cell lines were lysed in NP40 buffer (1% Nonidet P-40, 150 mM NaCl, 25 mM Tris-HCl pH 7.6, 1 mM DTT, 1 mM EDTA, 1 mM Sodium Orthovanadat, proteinase inhibitor (Roche, Lewes, UK)), sonicated for 30 sec and finally centrifuged for 30 min at 13.000 rpm. 20 μl of the supernatant (approximately 15-20 μg protein) was then mixed with 4x NuPage buffer (Invitrogen) and run on a 4-12% Bis-Tris Gel (Invitrogen).

Lysis of frozen tissue samples was processed according to the recommendations of the Femtelle Kit (FEMTELLE®). Approximately 100-150 mg cryoconserved tissue was homogenised with mortar and pestle. Next lysis was performed through addition of 2 ml lysis buffer (1,8 ml TBS (pH 8,5) + 0,2 ml 10% Triton-X-100) and centrifugation for 1 h at 100.000 g. 25 μl of the supernatant (approximately 15-20 μg protein) was then mixed with 4x NuPage buffer (Invitrogen) and run on a 4-12% Bis-Tris Gel (Invitrogen).

After SDS-PAGE (sodium dodecylsulfate polyacrylamide gel electrophoresis) the proteins were transferred to nitrocellulose. Blocking was performed with 5% nonfat dry milk overnight at 4°C. The primary antibody (SERBP1, Eurogentec) was then incubated for 1 h at room temperature at a dilution of 1:2000 for the cell line-lysates and 1:1000 for the tissue-lysates. Western blots were processed using a horseradish peroxidase goat anti-rabbit IgG (1:2000; Dako). The Pierce ECL Western Blotting Substrate was used for visualisation (Amersham Biosciences, Amersham, UK).

The same membrane was then used for development with a β-actin antibody (from mouse; Sigma) in a dilution of 1:2000. As secondary antibody a horseradish peroxidase goat anti- mouse IgG (1:2000; Dako) was used. As positive controls, tissue-lysates of liver and placenta were used. As a negative control rabbit IgG was used in place of the SERBP1 antibody.

The intensity ratio of SERBP1 protein expression was calculated densitometrically using a STORM-860 phosphoimager (Molecular Dynamics, Sunnyvale, CA, USA) and normalised against the β-actin expression.

### Statistical methods

For statistical evaluation the SPSS version 17.0 (SPSS Inc, Chicago, IL) was used. Differences were considered statistically significant when p-values were < 0.05. A non-parametrically two-tailed Mann-Whitney *U*-test was employed to analyse differences in expression levels. A statistical association between clinicopathological and molecular parameters was tested, using two-sided Fisher’s exact test. Recurrence-free (RFS) and overall survival (OS) were calculated according to the Kaplan-Meier equation.

For both TMAs, OS was defined as tumour-related death and RFS as any local recurrence or distant recurrence whatever occurred first. Simple Cox proportional hazard models were used with the biomarkers under investigation (nuclear and cytoplasmic IRS score of SERBP1) to analyse the prognostic effect with the biomarker as an ordinal variable. Kaplan-Meier estimates were used to display the survival curves and log rank test was used to compare patients with high vs. low SERBP1 expression. For multivariate analyses a Cox proportional hazard model was constructed for OS and RFS including tumour size (pT), nodal status (pN), Grading (G), ER status, PgR status and HER2/neu status. After analysing both datasets independently a joint analysis of the data was done for the adjusted hazard ratio with the same covariates

## Results

### SERBP1 mRNA is abundantly expressed in human normal breast tissue

**Figure 1:**
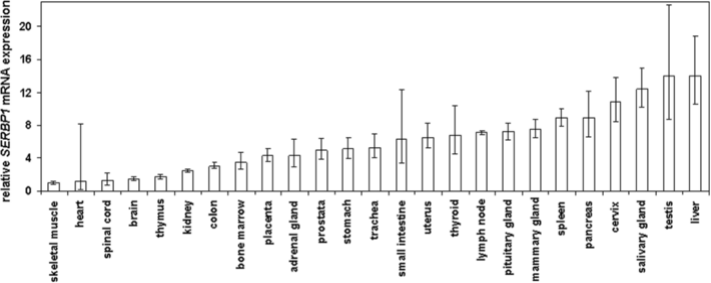
***SERBP1 *is abundantly expressed in human normal breast tissue.** Semiquantitative realtime PCR analysis of *SERBP1* expression was performed on reverse transcribed RNA from benign tissues derived from different human organs. *SERBP1* mRNA expression was low in the skeletal muscle, heart, spinal cord, brain and thymus whereas comparatively high expression levels were detected in the mammary gland, spleen, pancreas, cervix, salivary gland, testis and liver.

SERBP1 is a molecule which is thought to regulate the activity of the important breast cancer recurrence associated protein PAI-1 [2,23-26]. Recently, overexpression of SERBP1 was described in human ovarian cancer for the first time [1]. Interestingly, uPA and PAI-1 are also overexpressed in ovarian cancer and correlate with poor clinical outcome [12,13]. To analyse the *SERBP1* transcript level in human breast tissue we initiated our study by investigating the distribution of *SERBP1* mRNA expression in benign tissues derived from a variety of human organs. Real time PCR analysis showed that *SERBP1* is abundantly expressed in human normal breast tissue whereas relatively low levels could be detected in the skeletal muscle, heart, spinal cord, brain and thymus (Figure 1).

### SERBP1 is not differentially expressed between normal and malignant human breast tissue

**Figure 2:**
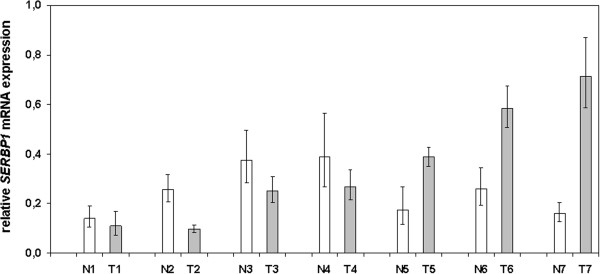
***SERBP1 *is not differentially expressed between normal and malignant human breast tissue in matched pairs.** A collection of cryoconserved matched pairs of normal (N; n = 7) and cancerous breast tissues (T; n = 7) was analysed for *SERBP1* expression by semiquantitative realtime PCR. *SERBP1* RNA was upregulated in three tumour tissues compared to the corresponding normal breast tissues. In four cancerous tissues *SERBP1* expression was lower than in the corresponding normal breast tissue. Expression levels between the respective groups were statistically not significant (*P* = 0.56).

We next analysed *SERBP1* mRNA expression in matched pairs of normal and malignant cryoconserved human breast tissue (Figure 2) to decipher whether *SERBP1* gets deregulated during breast cancer development as it has been shown in ovarian cancer [1]. In four breast cancer samples the rate of *SERBP1* mRNA was slightly lower than in the corresponding normal tissues. However *SERBP1* expression was slightly higher in three breast carcinomas compared with the matched normal breast specimens. In summary, *SERBP1* is not differentially expressed between normal and malignant human breast tissue and expression levels between the respective groups were statistically not significant (*P* = 0.56) (Figure 2). Additionally, *SERBP1* mRNA transcript levels of the cancerous breast tissues did neither correlate with the corresponding *PAI1* mRNA expression (*P* = 0.21) nor with the PAI-1 protein levels (*P* = 0.29) determined by ELISA test (FEMTELLE®) (data not shown).

### SERBP1 is expressed in breast epithelial cells and not in the stroma analysed by non-radioisotopic in situ hybridisation

**Figure 3:**
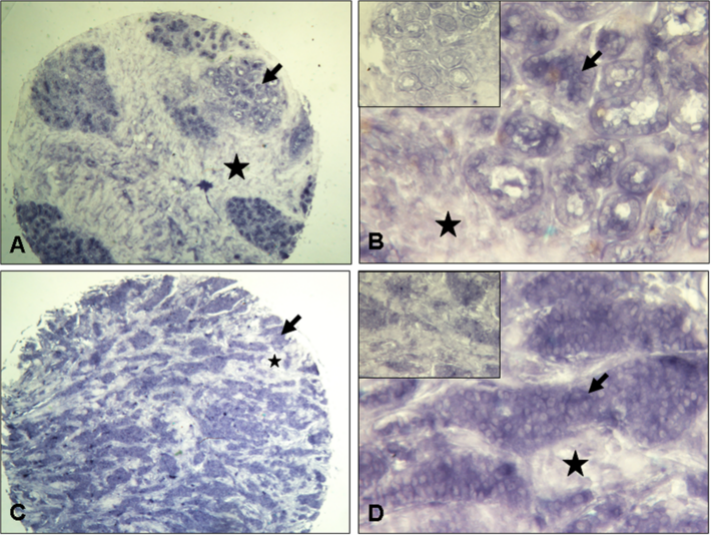
***SERBP1 *mRNA is expressed in breast epithelial cells but not in stromal cells as shown by non-radioisotopic *in situ *hybridisation SERBP1 expression was investigated by non-radioisotopic RNA *in situ *hybridisation in a subset of normal (n = 10) and cancerous (n = 10) breast tissue samples derived from the initial TMA set.** (**A**, **B**) Normal breast tissue with moderate SERBP1 mRNA expression in the epithelial cells (arrow) and no stromal (asterisk) staining using antisense SERBP1 RNA. (**B**) Insert shows no staining by SERBP1 sense RNA. (**C**, **D**) Invasive breast carcinoma (here: ductal type) also revealed moderate SERBP1 mRNA expression in the epithelial cells (arrow) applying antisense SERBP1 RNA whereas no staining could be seen in stromal cells (asterisk) (**D**) Insert shows negative staining by SERBP1 sense RNA. Magnifications: A, C: 40x; B, D: 200x.

*SERBP1* expression was not differentially expressed between normal and cancerous breast tissue as analyzed by realtime PCR analysis. To detect possible differential expression at the cellular level, we applied non-radioisotopic RNA in situ hybridisation in a subset of normal (n = 10) and cancerous (invasive ductal type, n = 10) breast tissue samples derived from the initial evaluation TMA. *SERBP1* mRNA was expressed in the benign and malignant breast epithelial cells but not in the stromal cells (Figure 3). However, also on the cellular level, *SERBP1* was not differentially expressed between normal and malignant breast tissue considering intensity and regional heterogeneity of the SERBP1 signal (Figure 3).

### SERBP1 antibody generation and validation by Western blot analysis using breast cell lines

**Figure 4:**
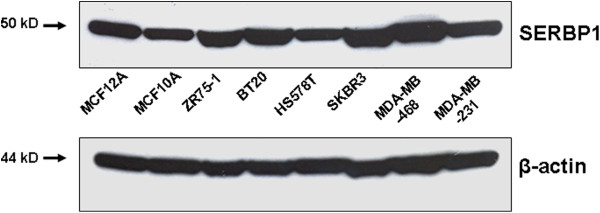
**SERBP1 antibody validation by Western blot analysis of breast cell lines.** The SERBP1 antibody was validated by Western blot analysis of benign (MCF12A, MCF10A) and malignant (ZR75-1, BT20, HS578T, SKBR3, MDA-MB468 and MDA-MB231) human mammary cell lines. Additionally, in line with the *SERBP1* mRNA data, SERBP1 protein expression in the normal mammary cell lines was not significantly different from the SERBP1 protein expression in the malignant mammary cell lines. β-actin displayed a homogenous protein loading in the investigated cell lines.

As we began our studies, commercial antibodies against SERBP1 were not available for immunohistochemistry. Therefore we generated a polyclonal SERBP1 antibody which was applicable for immunohistochemistry. The SERBP1 antibody was validated by Western blot analysis of benign (MCF12A, MCF10A) and malignant (ZR75-1, BT20, HS578T, SKBR3, MDA-MB468 and MDA-MB231) human mammary cell lines (Figure 4). As expected from our RNA expression data, we could detect a quite homogenous expression of SERBP1 protein in all cell lines. The calculated protein weight was approximately 50 kDa in all cell lysates. The slightly higher molecular weight of 50 kDa compared with the deduced molecular weight of 44965 Da (http://www.expasy.org/uniprot/Q8NC51#comments) was concordant with previous published data regarding Western blot analysis [6] and may be the result of posttranslational modification. The generated SERBP1 antibody was further validated by a peptide competition experiment using Western blot analysis (Additional file 1: Figure S1).

### SERBP1 protein is not differentially expressed between normal and cancerous human breast tissue analysing a tissue microarray

**Figure 5:**
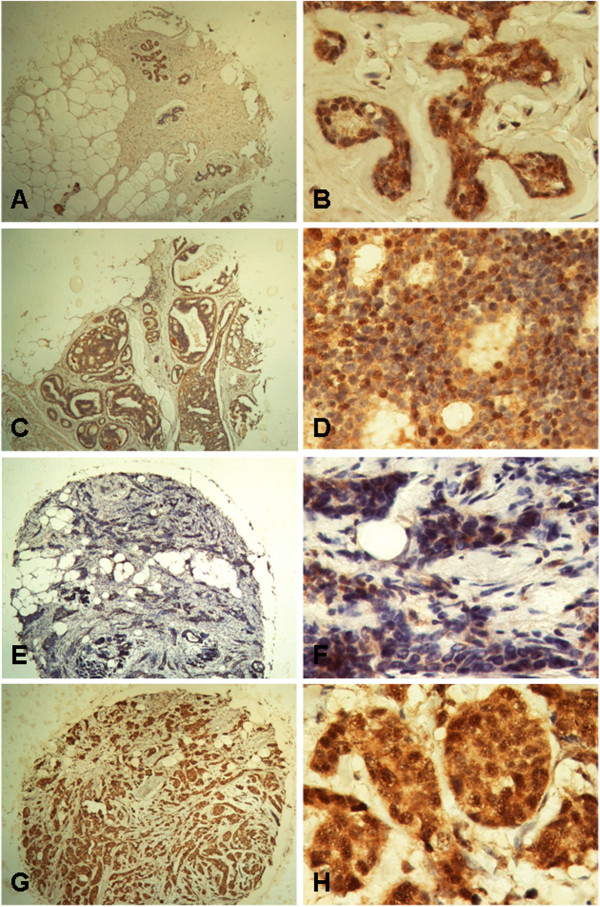
**SERBP1 protein is not differentially expressed between normal and cancerous human breast tissue on TMA.** SERBP1 protein was expressed heterogeneously in the nucleus as well as in the cytoplasm of normal and cancerous breast epithelial cells whereas no staining could be detected in stromal cells. Differences between the respective groups were statistically not significant. (**A**, **B**) Normal breast tissue showed moderate nuclear and cytoplasmic SERBP1 protein expression (IRS = 6) in the epithelial cells. (**C**, **D**) Ductal carcinoma *in situ* revealed a moderate nuclear and cytoplasmic SERBP1 staining (IRS = 6). (**E**, **F**) In invasive breast carcinoma (here: ductal type) SERBP1 expression was low (example shows nuclear and cytoplasmic staining with an IRS = 1). (**G**, **H**) Invasive breast carcinoma (here: ductal type) also displayed abundant nuclear (IRS = 8) and cytoplasmic SERBP1 protein expression (IRS = 12). Magnifications: A, C, E, G: 40x; B, D, F, H: 400x.

Next we applied the validated SERBP1 antibody on tissue microarrays. Two independent cohorts of breast cancer specimens arranged on two different tissue microarrays were analysed for SERBP1 expression by immunohistochemistry. These represented an initial evaluation set, consisting of 193 breast carcinomas and 48 normal breast tissues, and an independent validation set, consisting of 605 breast carcinomas. In normal breast tissue SERBP1 expression was heterogeneous with low to abundant nuclear and cytoplasmic staining in the epithelial cells whereas no staining could be seen in the stroma (Figure 5A and B). In ductal carcinoma in situ (Figure 5C and D) SERBP1 was expressed moderately in the nucleus as well as in the cytoplasm. In invasive breast carcinomas SERBP1 expression was generally very differential with low (Figure 5E and F, ductal type) to intense expression pattern in the nucleus as well as in the cytoplasm of the mammary epithelial cells (Figure 5G and H, ductal type). In whole, SERBP1 was expressed in both the nucleus and the cytoplasm of benign and malignant mammary epithelial cells and not in the stroma which is concordant with the results of the non-radioisotopic in situ hybridisation. In addition, SERBP1 expression was heterogeneous in normal and breast cancer specimens. Differences between the respective groups were statistically not significant which is in line with the *SERBP1* non-radioisotopic in situ hybridisation data, the *SERBP1* mRNA expression profile in tissues as well as with the Western blot analysis of SERBP1 protein expression in cell lines. The median immunoreactive scores (IRS) for nuclear (IRS = 6) and cytoplasmic (IRS = 4) staining were each identical between normal and cancerous breast tissues.

### Analysis of the initial evaluation TMA cohort

Data of the initial TMA cohort are presented in supplementary tables and figures (Additional file 2: Table S1, Additional file 3: Table S2, Additional file 1: Figure S1 and Additional file 4: Figure S2). In the initial TMA cohort, SERBP1 protein expression in breast carcinomas (IRS >2) was not associated with tumour size, lymph node status, histological grading, hormone receptor/Her2 status, focality or histological type of tumour (Additional file 2: Table S1). To investigate a possible impact of SERBP1 expression on patients’ clinical outcome we calculated univariate survival probability for nuclear and cytoplasmic SERBP1 IRS scores with respect to immunohistochemical results. We found that a higher SERBP1 expression (nuclear and cytoplasmic IRS score as ordinal variable) was nominally associated with a favourable prognosis for both, the nuclear staining and the cytoplasmic staining, regarding OS and RFS (p-values between 0.058 and 0.204), however none of the analyses reached statistical significance. The best cut-off in this cohort was a nuclear score greater than 2. The Kaplan-Meier analysis (*P* = 0.301) is shown in Additional file 3: Table S2 and Additional file 4: Figure S2. Patients who showed abundant SERBP1 expression in the tumour had an estimated mean RFS of 84 months (95% confidence interval: 74-94) compared to 67 months (95% confidence interval: 53-81) in patients with negative/weak SERBP1 expression. Although overall survival was also not significantly associated with abundant SERBP1 expression, there was a trend towards favourable prognosis regarding patients with strong SERBP1 expression (Additional file 3: Table S2).

### Validation in the Bavarian Breast Cancer Cases and Controls (BBCC) TMA cohort

To validate our results of the initial evaluation set of tumours, we further analysed a second larger cohort (BBCC) of breast cancer specimens on a TMA. In this validation cohort the IRS score was available for 605 invasive breast cancer cases. The mean patients’ age was 56 years (± 12). Chemotherapy was given to 48% of all patients and 92% of all hormone receptor positive patients received an antihormonal therapy. The median nuclear IRS score was 3 with 15% (n = 91) of tumours having a low score of 0 to 2 and 85% (n = 514) showing abundant expression of SERBP1 (IRS > 2). The cytoplasmic staining showed very similar results with a median score of 4 and 18% of the tumors having a core of 0 to 2 and 82% of the tumors having an abundant SERBP1 expression.

**Figure 6:**
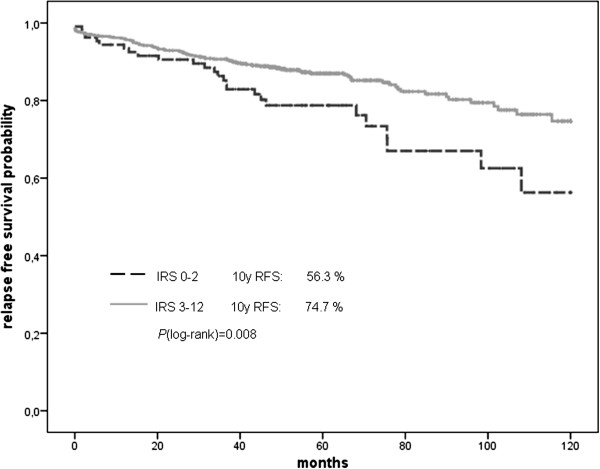
**Recurrence-free survival displayed as Kaplan Meier curves from the BBCC TMA.** Breast cancer patients with abundant SERBP1 expression showed favourable prognosis in recurrence-free survival analysis (*P* = 0.008).

**Figure 7:**
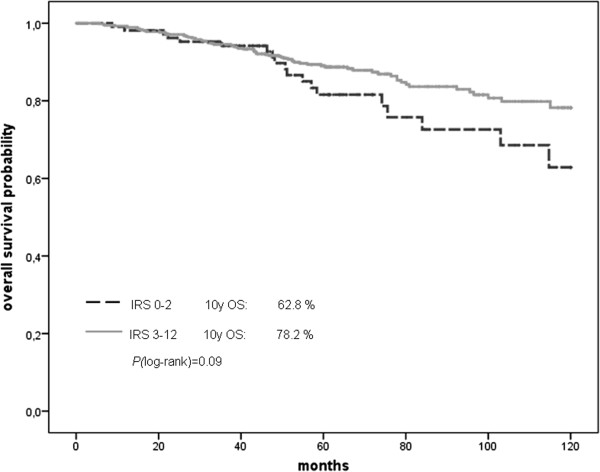
**Overall survival displayed as Kaplan Meier curves from the BBCC TMA.** Breast cancer patients overexpressing SERBP1 also exhibited favourable prognosis by trend in overall survival (*P* = 0.09).

In this larger cohort we found a significant correlation (*P* = 0.008) between nuclear SERBP1 expression and favourable prognosis in recurrence-free survival analysis (Figure 6). Although overall survival was not significantly associated with nuclear SERBP1 expression, we could see a trend towards favourable prognosis (*P* = 0.09) (Figure 7). In multivariate analysis, SERBP1 immunohistochemical expression turned out to be an independent predictor for RFS (HR = 0.59; 95% CI: 0.35 to 0.98, *P* = 0.039), but not for OS (HR = 0.79; 95% CI: 0.43 to 1.45) (Table 1). The cytoplasmic score showed no association with survival outcome (data not shown).

**Table 1:** Multivariate analysis (BBCC validation TMA) of recurrence-free survival, adjusted for pT, pN, Grading, ER, PR, HER2 and SERBP1

**Characteristic**		**N**	**Adjusted Hazard Ratio**	**95% CI**			**p-value**
pT^a^	1	312	1				
	2	227	1.85	1.10	to	3.10	0.02
	3	29	3.48	1.64	to	7.37	0.001
	4	37	7.25	3.81	to	13.80	1.7E-09
pN^a^	0	351	1				
	1	254	2.41	1.47	to	3.94	0.0005
Grading	1	49	1				
	2	361	1.40	0.43	to	4.57	0.57
	3	195	1.95	0.57	to	6.64	0.29
ER	Neg	143	1				
	Pos	462	1.19	0.63	to	2.24	0.59
PR	Neg	205	1				
	pos	400	0.58	0.33	to	1.04	0.067
HER2	Neg	511	1				
	pos	94	1.06	0.60	to	1.85	0.85
SERBP1	IRS 0-2	91	1				
	IRS 3-12	514	0.59	0.35	to	0.98	0.039

^a^According to Sobin LH, Wittekind CH: UICC: TNM Classification of Malignant Tumours. 6^th^ edition, New York: Wiley; 2002 [27].

pT: Pathological assessment of the primary tumour.

pN: Pathological assessment of the regional lymph nodes.

CI: Confidence interval.

Both datasets were analysed together to calculate the Cox proportional hazard ratios, adjusted for pT, pN, grading, ER/PR status, and HER2/neu status. The hazard ratio for recurrence-free survival gained in significance level with a HR = 0.64 (95%CI: 0.44 to 0.94; *P* = 0.02). The hazard ratio for overall survival remained not significant for SERBP1 with a HR of 0.92 (95%CI: 0.58 to 1.46; *P* = 0.73). Again the cytoplasmic score showed no association with survival outcome (data not shown).

### Abundant SERBP1 expression is associated with low PAI-1 protein level in Western blot analysis

**Figure 8:**
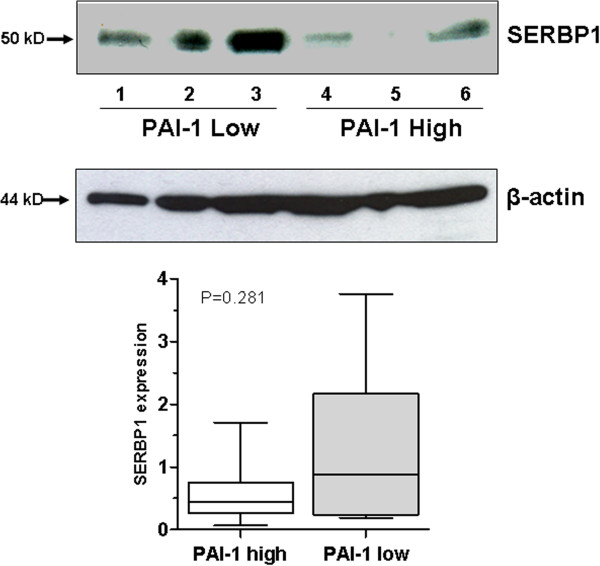
**Abundant SERBP1 expression is associated with low PAI-1 protein level in Western blot analysis.** SERBP1 protein expression was investigated in extracts of cryoconserved human breast cancer tissues with low (n = 8) and high (n = 8) levels of protein PAI-1 by using Western blot analysis. Extracts with low PAI-1 protein levels (number: 1-3) were associated with intense SERBP1 expression by trend and vice versa high PAI-1 protein levels (number: 4-6) correlated with low SERBP1 expression. Differences between the respective groups were statistically not significant (*P* = 0.281). β-actin showed a relatively homogenous protein loading in the investigated lysates.

Surprisingly, in the survival analyses abundant SERBP1 expression was associated with favourable prognosis which is inverse to the prognostic impact of the PAI-1 protein level because high PAI-1 protein levels are known to indicate unfavourable prognosis in human breast cancer [10,11]. Consequently we hypothesised that abundant SERBP1 protein expression might be related to low PAI-1 protein levels. Therefore we next determined SERBP1 protein expression in extracts of cryoconserved human breast cancer tissues with low (n = 8) and high (n = 8) levels of the protein PAI-1 as determined by the FEMTELLE® ELISA test. As we expected, extracts with low PAI-1 protein levels were associated with intense SERBP1 expression by trend. Vice versa high PAI-1 protein levels correlated with low SERBP1 expression although differences between the respective groups were statistically not significant (*P* = 0.281) (Figure 8). The mean densitometric intensity of SERBP1 expression in extracts with low PAI-1 protein levels was 1.31 arbitrary units (standard deviation (SD) ± 1.26) and 0.587 (SD ± 0.53) arbitrary units in extracts with high PAI-1 protein levels.

## Discussion

SERBP1 is a *PAI1 mRNA* binding protein that putatively regulates PAI-1 abundance by stabilising or destabilising PAI-1 *mRNA*[2]. Indeed, in rat hepatoma cells it could be shown that binding of SERBP1 protein to the *PAI1* mRNA leads to degradation and destabilisation of *PAI1* mRNA [3]. However, this interaction has not been analysed in human tissue including normal and cancerous breast tissue. In breast cancer, PAI-1 is associated with tumour invasion and metastasis, thus high uPA/PAI-1 protein levels are indicators of poor prognosis in this tumour entity [10,11]. Overexpression of PAI-1 has been reported in several human solid tumours besides breast cancer, including colorectal, gastric and cervical cancer [11,28-30]. Until now, SERBP1 overexpression was just shown in human ovarian carcinomas and was associated with advanced tumour stage [1]. In the current study, a systematic characterisation of SERBP1 expression in human breast cancer is presented for the first time at both the mRNA and the protein level, including a large cohort of breast carcinoma specimens that has been investigated by correlative analysis using clinicopathological parameters and patients’ survival data. We found that SERBP1 is not differentially expressed between normal and cancerous breast tissue as well as between benign and malignant breast epithelial cell lines. Differences in expression levels between the respective groups were statistically not significant which were underlined by the Western blot data of the benign and malignant mammary cell lines. These results were further encouraged by applying non-radioisotopic RNA *in situ* hybridisation on a subset of normal and cancerous breast tissues. Moreover, the lack of differential SERBP1 protein expression between benign and malignant breast samples could be confirmed by immunohistochemistry on a tissue microarray. Using this technique, SERBP1 protein expression was correlated with patients’ survival data. Interestingly, in our initial cohort of breast carcinomas (n = 193) we found a trend between abundant SERBP1 expression and favourable prognosis in recurrence-free survival (RFS; *P* = 0.301) pointing towards a potential protective role of SERBP1 in breast cancer development. Analysis of the second, independent breast cancer cohort (n = 605) confirmed this notion. In this validation TMA set, nuclear SERBP1 expression (IRS >2) was significantly correlated with favourable prognosis in recurrence-free survival analysis (*P* = 0.008) and turned out to be an independent prognostic marker in multivariate analysis. In overall survival analysis, nuclear SERBP1 expression was also associated with favourable prognosis (*P* = 0.09), but did not reach statistical significance. In summary, our survival analyses showed a correlation between nuclear SERBP1 expression and favourable prognosis which is inverse to the prognostic impact of PAI-1 as high PAI-1 protein levels are related to unfavourable prognosis in human breast cancer [10. 11]. An explanatory model for this inverse relationship between SERBP1 and PAI-1 could be the finding in rat hepatoma cells where high SERBP1 protein levels lead to degradation of PAI-1 mRNA and consecutively to low PAI-1 protein levels [3]. This explanation is encouraged by our Western blot analysis where breast carcinoma extracts with high PAI-1 protein levels showed weak SERBP1 protein expression by trend and vice versa breast carcinoma extracts with low PAI-1 protein levels showed abundant SERBP1 protein expression. However, this potential relationship between PAI-1 and SERBP1 protein levels has to be further validated in independent studies. Interestingly, we did not find a significant correlation between nuclear SERBP1 expression and nuclear progesterone receptor status in both TMAs as SERBP1 is known to mediate the membranous progesterone effect by binding to PGRMC1 [6]. The role of SERBP1 regarding the interaction with the nuclear progesterone receptor is unclear to date [31]. Progesterone receptors are classically defined as ligand-activated transcription factors, but also function at or near the plasma membrane to directly activate protein kinase pathways (namely, c-Src and the MAP kinase cascades consisting of Raf-1, MEK1/2 and ERK1/2). This is commonly called “rapid signalling”. The function of rapid signalling is unknown, but may provide positive regulation of PR-containing transcriptional complexes by direct phosphorylaton of PR or coregulatory molecules [25]. The relation between SERBP1 and progesterone receptor is quite important because progesterone receptors play a major role in the regulation of growth, survival and differentiation of normal and malignant breast epithelial cells [32,33]. Activated progesterone receptors are withheld in the nucleus and associate with numerous coregulatory molecules, including histone acetyl transferases, chromatin remodelling machines and TRAP/DRIP complexes that recruit RNA polymerase II [32]. Moreover, a positive progesterone receptor status is usually correlated with a positive oestrogen receptor status and favourable prognosis in human breast cancer [32,33]. However, the role of progesterone in the development of human breast cancer is poorly understood and controversially discussed [32,33]. Therefore inhibition of the progesterone receptor has not been established in the treatment of human breast cancer so far [32,33].

In summary, SERBP1 is a potential prognostic marker in human breast carcinoma. In further studies we are going to analyse the exact function of SERBP1 in the development of breast cancer and its interaction with the progesterone receptor and PAI-1 in this context.

## Conclusions

In conclusion, our analyses showed no differential SERBP1 expression between normal and cancerous human breast tissue both at the RNA and protein level. Surprisingly, nuclear SERBP1 expression in breast carcinoma was significantly associated with favourable prognosis in recurrence-free survival analysis which is inverse to the prognostic impact of the proteins uPA/PAI-1. An explanation for this inverse relationship might be the degradation of *PAI*-*1* mRNA by binding of the SERBP1 protein to it as it was described in rat hepatoma cells previously and which was underlined by our Western blot analyses by trend. Furthermore, SERBP1 may represent a novel breast tumour marker with prognostic significance. However, the putative function of SERBP1 in breast carcinogenesis and its relation to PAI-1 has to be further analysed in prospective studies.

## Abbreviations

SERBP1: PAI-RBP1 = *PAI-1* mRNA binding protein; ER: Oestrogen receptor; PR: Progesterone receptor; PGRMC1: Progesterone receptor membrane component-1; RT-PCR: Reverse transcription – polymerase chain reaction; GAPDH: Glyeraldehyde-3-phosphate dehydrogenase; RFS: Recurrence-free survival; OS: Overall survival; TMA: Tissue microarray; IRS: Immunoreactivity Score; BBCC: Bavarian Breast Cancer Cases and Controls Study.

## Competing interests

The authors declare that they have no competing interests.

## Authors’ contributions

NBS: participated in design of the study, data analysis, data interpretation, establishment and evaluation of the immunohistochemistry and drafted the manuscript; AB: carried out the RT-PCR studies, supported with expertise in molecular biology techniques and data interpretation; IK: performed the non-radioisotopic *in situ* hybridisation and participated in data analysis; SvS: carried out the immunohistochemistry and Western blot analyses and critically revised the manuscript; EN: supported in data analysis, interpretation and critically revised the manuscript; MFP: participated in construction of the BBCC-TMA and data analysis; AD: participated in construction of the BBCC-TMA and data analysis; AH: provided the initial TMA set, supported in data interpretation and critically revised the manuscript; JS: participated in antibody generation and data analysis; MWB: supported in data interpretation and critically revised the manuscript; RK: supported in data interpretation and critically revised the manuscript; PAF: participated in construction of the BBCC-TMA, study design and coordination, data analysis, data interpretation and drafting of the manuscript; ED conceived the study, participated in study design and coordination, molecular and data analysis, data interpretation and drafting of the manuscript. All authors read and approved the final manuscript.

## Pre-publication history

The pre-publication history for this paper can be accessed here:

http://www.biomedcentral.com/1471-2407/12/597/prepub

## Supplementary Material

Additional file 1 **Figure S1.** Validation of SERBP1 antibody in Western blot analysis/Competition test. The SERBP1 antibody was validated in Western blot analysis by using the peptide competition test. SERBP1 protein expression was investigated in extracts of cryoconserved placenta, liver and in the breast cancer cell lines MDA-MB 231 and BT20. After peptide competition the SERBP1 protein signal was absent or weak whereas ß-actin displayed a homogenous protein loading. Click here for file

Additional file 2 **Table S1.** Clinicopathological and immunohistochemical parameters in relation to SERBP1 immunoreactivity in the evaluation TMA. Click here for file

Additional file 3 **Table S2.** Univariate analysis of factors regarding overall survival (OS) and recurrence-free survival (RFS) in the evaluation TMA. Click here for file

Additional file 4 **Figure S2.** Correlation of SERBP1 expression and patient prognosis according to univariate Kaplan-Meier analysis. Breast cancer patients overexpressing SERBP1 presented favourable prognosis in recurrence-free survival analysis by trend (*P* = 0.301). Click here for file

## References

[B1] Koensgen D Mustea A Klaman I Sun P Zafrakas M Lichtenegger W Denkert C Dahl E Sehouli J Expression analysis and RNA localization of PAI-RBP1 (SERBP1) in epithelial ovarian cancer: association with tumor progression Gynecol Oncol 2007 107 266 273 10.1016/j.ygyno.2007.06.023 17698176

[B2] Heaton JH Dlakic WM Dlakic M Gelehrter TD Identification and cDNA cloning of a novel RNA-binding protein that interacts with the cyclic nucleotide-responsive sequence in the Type-1 plasminogen activator inhibitor mRNA J Biol Chem 2001 276 3341 3347 10.1074/jbc.M006538200 11001948

[B3] Heaton JH Dlakic WM Gelehrter TD Posttranscriptional regulation of PAI-1 gene expression Thromb Haemost 2003 89 959 966 Review. 12783107

[B4] Lemos TA Passos DO Nery FC Kobarg J Characterization of a new family of proteins that interact with the C-terminal region of the chromatin-remodeling factor CHD-3 FEBS Lett 2003 533 14 20 12505151 10.1016/s0014-5793(02)03737-7

[B5] Felsenfeld G Chromatin as an essential part of the transcriptional mechanism Nature 1992 355 219 224 Review. 10.1038/355219a0 1731219

[B6] Peluso JJ Romak J Liu X Progesterone receptor membrane component-1 (PGRMC1) is the mediator of progesterone’s antiapoptotic action in spontaneously immortalized granulosa cells as revealed by PGRMC1 small interfering ribonucleic acid treatment and functional analysis of PGRMC1 mutations Endocrinology 2008 149 534 543 17991724 10.1210/en.2007-1050 PMC2219306

[B7] Andreasen PA Kjøller L Christensen L Duffy MJ The urokinase-type plasminogen activator system in cancer metastasis: a review Int J Cancer 1997 72 1 22 Review. 10.1002/(SICI)1097-0215(19970703)72:1<1::AID-IJC1>3.0.CO;2-Z 9212216

[B8] Schmitt M Harbeck N Thomssen C Wilhelm O Magdolen V Reuning U Ulm K Höfler H Jänicke F Graeff H Clinical impact of the plasminogen activation system in tumor invasion and metastasis: prognostic relevance and target for therapy Thromb Haemost 1997 78 285 296 Review. 9198168

[B9] Rosenberg S The urokinase-type plasminogen activator system in cancer and other pathological conditions: introduction and perspective Curr Pharm Des 2003 9 4p Review. 10.2174/138161281030900iv 12877116

[B10] Zemzoum I Kates RE Ross JS Dettmar P Dutta M Henrichs C Yurdseven S Höfler H Kiechle M Schmitt M Harbeck N Invasion factors uPA/PAI-1 and HER2 status provide independent and complementary information on patient outcome in node-negative breast cancer J Clin Oncol 2003 21 1022 1028 10.1200/JCO.2003.04.170 12637466

[B11] Harbeck N Thomssen C 2003 Zentralbl Gynakol 2003 125 362 367 14569518 10.1055/s-2003-43036

[B12] Kuhn W Schmalfeldt B Reuning U Pache L Berger U Ulm K Harbeck N Späthe K Dettmar P Höfler H Jänicke F Schmitt M Graeff H Prognostic significance of urokinase (uPA) and its inhibitor PAI-1 for survival in advanced ovarian carcinoma stage FIGO IIIc Br J Cancer 1999 79 1746 1751 10.1038/sj.bjc.6690278 10206287 PMC2362775

[B13] Tecimer C Doering DL Goldsmith LJ Meyer JS Abdulhay G Wittliff JL Clinical relevance of urokinase-type plasminogen activator, its receptor and inhibitor type 1 in ovarian cancer Int J Gynecol Cancer 2000 10 372 381 10.1046/j.1525-1438.2000.010005372.x 11240701

[B14] Dahl E Kristiansen G Gottlob K Klaman I Ebner E Hinzmann B Hermann K Pilarsky C Durst M Klinkhammer-Schalke M Blaszyk H Knuechel R Hartmann A Rosenthal A Wild PJ Molecular profiling of laser-microdissected matched tumor and normal breast tissue identifies karyopherin alpha2 as a potential novel prognostic marker in breast cancer Clin Cancer Res 2006 12 3950 3960 10.1158/1078-0432.CCR-05-2090 16818692

[B15] Bektas N Noetzel E Veeck J Press MF Kristiansen G Naami A Hartmann A Dimmler A Beckmann MW Knüchel R Fasching PA Dahl E The ubiquitin-like molecule interferon-stimulated gene 15 (ISG15) is a potential prognostic marker in human breast cancer Breast Cancer Res 2008 10 4 R58 Epub 2008 Jul 15. 10.1186/bcr2117 18627608 PMC2575531

[B16] Elston CW Ellis IO Pathological prognostic factors in breast cancer. The value of histological grade in breast cancer: experience from a large study with long-term follow-up. Histopathology 1991 19 403 410 10.1111/j.1365-2559.1991.tb00229.x 1757079

[B17] Fasching PA Loehberg CR Strissel PL Lux MP Bani MR Schrauder M Geiler S Ringleff K Oeser S Weihbrecht S Schulz-Wendtland R Hartmann A Beckmann MW Strick R Single nucleotide polymorphisms of the aromatase gene (CYP19A1), HER2/neu status, and prognosis in breast cancer patients Breast Cancer Res Treat 2007 epub ahead of print: DOI 10.1007/s10549-007-9822-2 10.1007/s10549-007-9822-2 18049890

[B18] Schrauder M Frank S Strissel PL Lux MP Bani MR Rauh C Sieber CC Heusinger K Hartmann A Schulz-Wendtland R Strick R Beckmann MW Fasching PA Single nucleotide polymorphism D1853N of the ATM gene may alter the risk for breast cancer J Cancer Res Clin Oncol 2008 134 873 882 10.1007/s00432-008-0355-9 18264724 PMC12160744

[B19] Veeck J Niederacher D An H Klopocki E Wiesmann F Betz B Galm O Camara O Durst M Kristiansen G Huszka C Knuchel R Dahl E Aberrant methylation of the Wnt antagonist SFRP1 in breast cancer is associated with unfavourable prognosis Oncogene 2006 25 3479 3488 10.1038/sj.onc.1209386 16449975

[B20] Chekerov R Klaman I Zafrakas M Könsgen D Mustea A Petschke B Lichtenegger W Sehouli J Dahl E Altered expression pattern of topoisomerase IIalpha in ovarian tumor epithelial and stromal cells after platinum-based chemotherapy Neoplasia 2006 8 38 45 10.1593/neo.05580 16533424 PMC1584288

[B21] Veeck J Dahl E RNA expression analysis on formalin-fixed paraffin-embedded tissues in TMA format by RNA in situ hybridization Methods Mol Biol 2010 664 135 150 10.1007/978-1-60761-806-5_14 20690060

[B22] Remmele W Stegner HE Recommendation for uniform definition of an immunoreactive score (IRS) for immunohistochemical estrogen receptor detection (ER-ICA) in breast cancer tissue Pathologe 1987 8 138 140 3303008

[B23] Harbeck N Kates RE Look MP Meijer-Van Gelder ME Klijn JG Krüger A Kiechle M Jänicke F Schmitt M Foekens JA Enhanced benefit from adjuvant chemotherapy in breast cancer patients classified high-risk according to urokinase-type plasminogen activator (uPA) and plasminogen activator inhibitor type 1 (n = 3424) Cancer Res 2002 62 16 4617 4622 12183417

[B24] Harbeck N Kates RE Schmitt M Clinical relevance of invasion factors urokinase-type plasminogen activator and plasminogen activator inhibitor type 1 for individualized therapy decisions in primary breast cancer is greatest when used in combination J Clin Oncol 2002 20 4 1000 1007 10.1200/JCO.20.4.1000 11844823

[B25] Harbeck N Thomssen C Berger U Ulm K Kates RE Höfler H Jänicke F Graeff H Schmitt M Invasion marker PAI-1 remains a strong prognostic factor after long-term follow-up both for primary breast cancer and following first relapse Breast Cancer Res Treat 1999 54 2 147 157 10.1023/A:1006118828278 10424405

[B26] Harris L Fritsche H Mennel R Norton L Ravdin P Taube S Somerfield MR Hayes DF Bast RC Jr American Society of Clinical Oncology American Society of Clinical Oncology 2007 update of recommendations for the use of tumor markers in breast cancer J Clin Oncol 2007 25 33 5287 5312 10.1200/JCO.2007.14.2364 17954709

[B27] Sobin LH Wittekind CH UICC TNM Classification of Malignant Tumours 2002 6 New York: Wiley

[B28] Berger DH Plasmin/plasminogen system in colorectal cancer World J Surg 2002 26 767 771 10.1007/s00268-002-4050-8 11965442

[B29] Nekarda H Siewert JR Schmitt M Ulm K Tumour-associated proteolytic factors uPA and PAI-1 and survival in totally resected gastric cancer Lancet 1994 343 117 10.1016/S0140-6736(94)90845-1 7903748

[B30] Kobayashi H Fujishiro S Terao T Impact of urokinase-type plasminogen activator and its inhibitor type 1 on prognosis in cervical cancer of the uterus Cancer Res 1994 54 6539 6548 7987854

[B31] Lee YJ Hsieh WY Chen LY Li C Protein arginine methylation of SERBP1 by protein arginine methyltransferase 1 affects cytoplasmic/nuclear distribution J Cell Biochem 2012 113 8 2721 2728 10.1002/jcb.24151 22442049

[B32] Lange CA Yee D Progesterone and breast cancer Womens Health (Lond Engl) 2008 4 151 162 10.2217/17455057.4.2.151 19072517 PMC4038907

[B33] Lange CA Sartorius CA Abdel-Hafiz H Spillman MA Horwitz KB Jacobsen BM Progesterone receptor action: translating studies in breast cancer models to clinical insights Adv Exp Med Biol 2008 630 94 111 10.1007/978-0-387-78818-0_7 18637487

