# Supplementary figures and images for: Overexpression of SERBP1 (Plasminogen activator inhibitor 1 RNA binding protein) in human breast cancer is correlated with favourable prognosis
Source: BMC Cancer. 2012 Dec 13;12:597. doi: 10.1186/1471-2407-12-597 (PMC3538721; doi:10.1186/1471-2407-12-597)

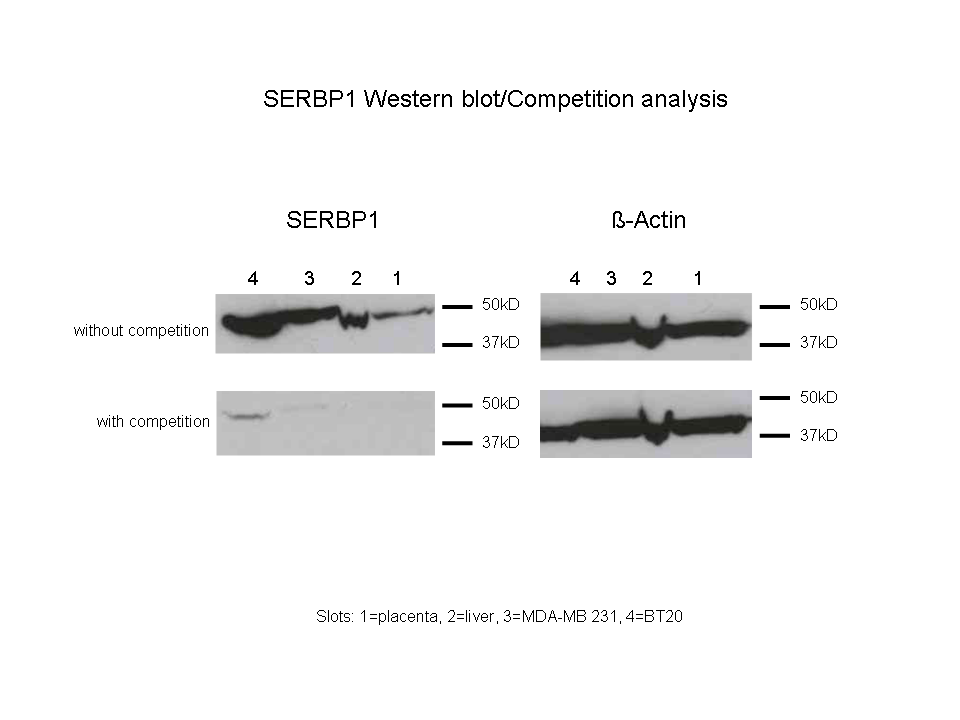

Supplement: Additional file 1 — Figure S1. Validation of SERBP1 antibody in Western blot analysis/Competition test. The SERBP1 antibody was validated in Western blot analysis by using the peptide competition test. SERBP1 protein expression was investigated in extracts of cryoconserved placenta, liver and in the breast cancer cell lines MDA-MB 231 and BT20. After peptide competition the SERBP1 protein signal was absent or weak whereas ß-actin displayed a homogenous protein loading. [file 1471-2407-12-597-S1.tiff]

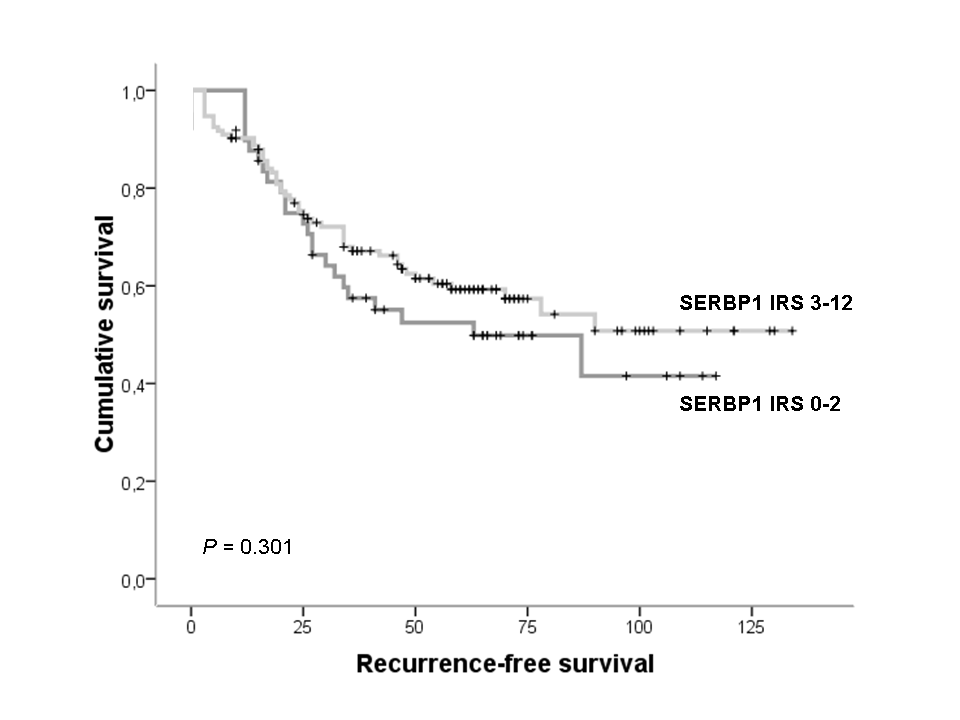

Supplement: Additional file 4 — Figure S2. Correlation of SERBP1 expression and patient prognosis according to univariate Kaplan-Meier analysis. Breast cancer patients overexpressing SERBP1 presented favourable prognosis in recurrence-free survival analysis by trend (P = 0.301). [file 1471-2407-12-597-S4.tiff]
