# Supplementary material for: Overexpression of SERBP1 (Plasminogen activator inhibitor 1 RNA binding protein) in human breast cancer is correlated with favourable prognosis
Source: BMC Cancer. 2012 Dec 13;12:597. doi: 10.1186/1471-2407-12-597 (PMC3538721; doi:10.1186/1471-2407-12-597)
Supplement: Additional file 2 — Table S1. Clinicopathological and immunohistochemical parameters in relation to SERBP1 immunoreactivity in the evaluation TMA. [file 1471-2407-12-597-S2.doc]

| **Supp. Table 1.** Clinicopathological and immunohistochemical parameters | | | | | |
| --- | --- | --- | --- | --- | --- |
| in relation to SERBP1 immunoreactivity in the evaluation TMA | | | | | |
|  |  |  |  |  |  |
| **Variable** | **Categorisation** | **SERBP1 immunoreactivity** | | | |
|  |  | **n analysable** | **low**^b^ | **abundant**^b^ | **p**^c^ |
|  |  |  |  |  |  |
| ***Clinicopathological data:*** | |  |  |  |  |
| Tumour stage^a^ | |  |  |  |  |
|  | pT1 | 53 | 15 | 38 | 0.146 |
|  | pT2 | 94 | 28 | 66 |  |
|  | pT3 | 13 | 0 | 13 |  |
|  | pT4 | 29 | 9 | 20 |  |
| Lymph node status^a^ | |  |  |  |  |
|  | pN0 | 81 | 20 | 61 | 0.625 |
|  | pN1-3 | 104 | 29 | 75 |  |
| Histological grade | |  |  |  |  |
|  | G1 | 19 | 7 | 12 | 0.604 |
|  | G2 | 88 | 23 | 65 |  |
|  | G3 | 81 | 21 | 60 |  |
| Multifocality | |  |  |  |  |
|  | unifocal tumour | 161 | 47 | 114 | 0.184 |
|  | multifocal tumour | 29 | 5 | 24 |  |
| Histological type | |  |  |  |  |
|  | ductal | 152 | 40 | 112 | 0.829 |
|  | lobular | 16 | 5 | 11 |  |
|  | other | 19 | 6 | 13 |  |
|  |  |  |  |  |  |
| ***Immunohistochemistry (IHC):*** | |  |  |  |  |
| Oestrogen receptor status | |  |  |  |  |
|  | negative (IRS 0-2) | 50 | 15 | 35 | 0.410 |
|  | positive (IRS 3-12) | 105 | 25 | 80 |  |
| Progesterone receptor status | |  |  |  |  |
|  | negative (IRS 0-2) | 111 | 28 | 83 | 0.673 |
|  | positive (IRS 3-12) | 54 | 12 | 42 |  |
| HER2 status | |  |  |  |  |
|  | weak (0-2+) | 135 | 38 | 97 | 0.078 |
|  | strong (3+) | 31 | 4 | 27 |  |
|  |  |  |  |  |  |
| ^a^According to UICC: TNM Classification of Malignant Tumours. 6^th^ edn (2002) Sobin  LH, Wittekind CH (eds) Wiley: New York [32] | | | | | |
| ^b^SERBP1 immunoreactivity: low=IRS 0-2, abundant=IRS 3-12 | | | | | |
| ^c^Pearson test (two-sided), bold face representing significant data (*P* < 0.05) | | | | | |
