# Supplementary material for: Overexpression of SERBP1 (Plasminogen activator inhibitor 1 RNA binding protein) in human breast cancer is correlated with favourable prognosis
Source: BMC Cancer. 2012 Dec 13;12:597. doi: 10.1186/1471-2407-12-597 (PMC3538721; doi:10.1186/1471-2407-12-597)
Supplement: Additional file 3 — Table S2. Univariate analysis of factors regarding overall survival (OS) and recurrence-free survival (RFS) in the evaluation TMA. [file 1471-2407-12-597-S3.doc]

| **Supp. Table 2.** Univariate analysis of factors regarding | | | | | | | | | | |
| --- | --- | --- | --- | --- | --- | --- | --- | --- | --- | --- |
| overall survival (OS) and recurrence-free survival (RFS) in the evaluation TMA | | | | | | | | | | |
|  |  |  | | |  | | |  | |  |
| **Variable** | **Categorisation** | **Tumour-related death (OS)** | | | | **Tumour recurrence (RFS)** | | | | |
|  |  | **n** | **events** | **p**^c^ | | **n** | **events** | | **p**^c^ | |
|  |  |  |  |  | |  |  | |  | |
| ***Clinicopathological data:*** | |  |  |  | |  |  | |  | |
| Tumour stage^a^ | |  |  |  | |  |  | |  | |
|  | pT1 | 53 | 8 | **<0.0001** | | 51 | 11 | | **<0.0001** | |
|  | pT2 | 94 | 32 |  |  | 91 | 42 | |  |  |
|  | pT3 | 13 | 3 |  |  | 12 | 6 | |  |  |
|  | pT4 | 29 | 20 |  |  | 26 | 18 | |  |  |
| Lymph node status^a^ | |  |  |  | |  |  | |  | |
|  | pN0 | 81 | 11 | **<0.0001** | | 79 | 16 | | **<0.0001** | |
|  | pN1-3 | 104 | 48 |  |  | 100 | 58 | |  |  |
| Histological grade | |  |  |  | |  |  | |  | |
|  | G1 | 19 | 5 | **0.002** | | 18 | 5 | | **0.001** | |
|  | G2 | 88 | 21 |  |  | 83 | 27 | |  |  |
|  | G3 | 81 | 37 |  |  | 79 | 44 | |  |  |
| Multifocality | |  |  |  | |  |  | |  | |
|  | unifocal tumour | 161 | 53 | 0.849 | | 154 | 65 | | 0.918 | |
|  | multifocal tumour | 29 | 10 |  |  | 27 | 12 | |  |  |
| Histological type | |  |  |  | |  |  | |  | |
|  | ductal | 152 | 50 | 0.641 | | 148 | 66 | | 0.225 | |
|  | lobular | 16 | 7 |  |  | 14 | 5 | |  |  |
|  | other | 19 | 5 |  |  | 17 | 4 | |  |  |
|  |  |  |  |  | |  |  | |  | |
| ***Immunohistochemistry (IHC):*** | |  |  |  | |  |  | |  | |
| Oestrogen receptor status | |  |  |  | |  |  | |  | |
|  | negative (IRS 0-2) | 50 | 20 | 0.096 | | 50 | 25 | | 0.107 | |
|  | positive (IRS 3-12) | 105 | 30 |  |  | 100 | 35 | |  |  |
| Progesterone receptor status | |  |  |  | |  |  | |  | |
|  | negative (IRS 0-2) | 111 | 47 | **<0.001** | | 104 | 51 | | **0.014** | |
|  | positive (IRS 3-12) | 54 | 9 |  |  | 54 | 15 | |  |  |
| HER2 status | |  |  |  | |  |  | |  | |
|  | weak (0-2+) | 135 | 38 | **0.005** | | 127 | 49 | | **0.039** | |
|  | strong (3+) | 31 | 16 |  |  | 31 | 17 | |  |  |
| SERBP1^b^ |  |  |  |  | |  |  | |  | |
|  | low (IRS 0-2) | 52 | 17 | 0.824 | | 49 | 24 | | 0.301 | |
|  | abundant (IRS 3-12) | 139 | 46 |  |  | 133 | 53 | |  |  |
|  |  |  |  |  | |  |  | |  | |
| ^a^According to UICC: TNM Classification of Malignant Tumours. 6^th^ edn (2002) Sobin LH, Wittekind CH (eds) Wiley: New York [32] | | | | | | | | | | |
| ^b^SERBP1 immunoreactivity: low=IRS 0-2, abundant=IRS 3-12 | | | | | | | | | | |
| ^c^Log-rank test (two-sided), bold face representing significant data (*P* < 0.05) | | | | | | | | | | |
